# Expression of HER3, HER4 and their ligand heregulin-4 is associated with better survival in bladder cancer patients

**DOI:** 10.1038/sj.bjc.6602251

**Published:** 2004-12-07

**Authors:** A A Memon, B S Sorensen, P Melgard, L Fokdal, T Thykjaer, E Nexo

**Affiliations:** 1 Department of Clinical Biochemistry, AKH University Hospital, Norrebrogade 44, 8000 Aarhus C, Denmark; 2 Department of Oncology, AKH University Hospital, Norrebrogade 44, 8000 Aarhus C, Denmark; 3 Department of Clinical Biochemistry, Skejby Hospital, 8200 Aarhus N, Denmark

**Keywords:** HER3, HER4, heregulins, patient survival, bladder cancer

## Abstract

The epidermal growth factor system has been associated to prognosis in patients with bladder cancer based mainly on the expression of the epidermal growth factor (EGF) receptor 1 (EGFR) and HER2 and their activating ligands. Since limited information exists concerning the expression of other parts of the EGF system, we examined the expression of the receptors HER3 and HER4 and their activating ligands, the heregulins (HRGs), in bladder cancer patients. Biopsies from bladder cancer tumours were obtained from 88 patients followed for a median of 23 months (range, 1–97 months). The mRNA content of four ligands and their isoforms (HRG1*α*, HRG1*β*, HRG2*α*, HRG2*β*, HRG3 and HRG4) and two receptors (HER3 and HER4) was quantified by real-time PCR. A significantly lower mRNA expression level of HER3 (*P*=0.0003), HRG2*α* (*P*=0.0159), HRG2*β* (*P*=0.0007) and HRG4 (*P*<0.0001) was observed in muscle-invasive (T2–T4) tumours as compared to superficial (Ta) tumours. The expression of HER3 mRNA correlated strongly to overall survival (*P*=0.0042); increased expression of HER4 (*P*=0.0261) and HRG4 (*P*=0.0245) was also associated with better prognosis. Interestingly, patients with coexpression of HER3 (*P*=0.0034) or HER4 (*P*=0.0080) together with their stimulating ligand HRG4 showed even better survival than for HER3 or HER4 alone. Our results together with previous data suggest a dual face for the EGF system. While it is well established that an increased signalling through HER1 and HER2 is related to a poor prognosis, our data suggest that signalling through HER3 and HER4 is related to a favourable outcome in bladder cancer patients.

Bladder cancer is the fifth most common cancer in men and ninth in women (Droller, 1998). Despite much research on the topic, it is still difficult to predict tumour progression, optimal therapy and clinical outcome (Stein *et al*, 1998; Burchardt *et al*, 2000). Tumour staging is considered to be the best prognostic marker, but several other markers including the presence of members of the epidermal growth factor (EGF) family have been suggested (Kristensen *et al*, 1988; Mellon *et al*, 1996; Ravery *et al*, 1997; Vollmer *et al*, 1998).

The EGF family consists of four receptors, HER1 (human EGF receptor 1, also known as EGFR, HER2 (ErbB2), HER3 (ErbB3) and HER4 (ErbB4). For HER2, no ligand has so far been described, and HER3 is characterised by its impaired kinase function (Guy *et al*, 1994). The ligands for these receptors consist of approximately 20 different proteins encoded by at least 10 different genes (Gullick, 2001; Yarden and Sliwkowski, 2001). The numerous EGF family-specific ligands include EGF and five other ligands able to bind to HER1, whereas the heregulins (HRGs) are the ligands for HER3 and HER4. Currently there are four known HRG genes, HRG1, HRG2, HRG3 and HRG4. HRG1 and HRG2 include multiple splicing isoforms, and these are denoted as either alpha or beta isoforms depending on the sequence of the EGF homology domain, which is a sequence element conserved among all the HRGs and characterised by its homology to sequences in EGF.

Previous studies have indicated that the EGF system plays an important role in bladder cancer. Among the four EGF receptors, HER1, HER2 and their activating ligands show a positive correlation with tumour stage in transitional carcinoma of the bladder (Neal *et al*, 1985; Messing *et al*, 1987; Thogersen *et al*, 2001). The same holds for a number of other types of malignancies, which within the last years has led to the development of a new treatment strategy aimed to inhibit signalling through HER1 or HER2 (de Bono and Rowinsky, 2002; Kubo *et al*, 2003; Lammering *et al*, 2003; Rowinsky, 2003). However, the role of HER3 and HER4 in human tumours is at present unclear. Conflicting reports on this topic have been published, as some have emphasised that HER3 and HER4 correlate with poor prognosis in human breast cancer (Bieche *et al*, 2003), while others have shown that HER3 and HER4 elevated expressions are associated with a better prognosis in this disease (Pawlowski *et al*, 2000). However, in the case of HER4, the few clinical studies carried out so far have suggested that its expression is associated with favourable prognosis (Thybusch-Bernhardt *et al*, 2001; Suo *et al*, 2002) and recently it has been reported that HER4 mediates ligand-dependent antiproliferative and differentiation responses in human breast cancer cells (Earp *et al*, 2003). The binding of HRG to its receptors induces either HER3 or HER4 to form homodimers or heterodimers mostly with HER2, thus triggering diverse signalling cascades (Bacus *et al*, 1992). Heregulin can regulate a variety of responses in cultured cells, including proliferation, differentiation and survival (Burden and Yarden, 1997). In AU565 and MDA-MB-453 human breast cancer cells, a low concentration of HRG is mitogenic, whereas a higher concentration leads to differentiation and inhibition of cell growth (Bacus *et al*, 1992). Current studies suggest that HRGs also initiate programmed cell death and induce cell differentiation (Le *et al*, 2000; 2002). Although much progress has been achieved in understanding HRG-induced signalling, the expression of HRGs and their prognostic significance in cancer patients has not been examined, most likely because of limitation in available methods.

Epidermal growth factor family gene expression can reliably be studied at the mRNA level, and based on a number of studies the expression is likely to reflect the presence of the corresponding protein (Knowlden *et al*, 1998; Walker and Dearing, 1999; Suo *et al*, 2002; Junttila *et al*, 2003).

In this report, we investigate the mRNA expression of HER3, HER4, HRG1, HRG2, HRG3 and HRG4 genes, including the splice variants from HRG1 and HRG2, in biopsies from 88 bladder cancer patients using the real-time RT–PCR method. In contrast to HER1 and HER2 and their activating ligands (Thogersen *et al*, 2001), we report that an increased expression of HER3 and HER4 and their activating ligands is related to a favourable prognosis.

## MATERIALS AND METHODS

### Patients

A total of 88 patients with primary bladder cancer were included. Biopsies were obtained by transurethral tumour resection. Tumour stage was assigned according to the Union Internationale Contre le Cancer Tumor-Node-Metastasis system (Spiessl *et al*, 2003). Grading was performed in accordance with the methods described by Bergkvist *et al* (1965). The relationships between the distribution of tumour stage, age and sex are presented in Table 1

**Table 1:** Clinical data

**Tumour stage**	**Ta**	**T1**	**T2–T4**
Patient (no.)	21	18	49
Sex			
Male	11	17	41
Female	10	1	8
Age			
Median	69	74	68
Range	53–88	60–88	54–83

. Patients were allocated into three groups depending on tumour stage: Ta, superficial tumours; T1, superficial invasive tumours; and T2–T4, muscle-invasive tumours. At the time of inclusion, 18 patients had received treatment in the form of radical radiotherapy, chemotherapy or intravesical therapy with bacillus Calmette–Guerin. The follow-up period was from the date of biopsy to the day of death or to December 2003. Patients were censored if they were alive at the time of analysis (December 2003). The median follow-up was 23 months (range, 1–97 months). The regional committee of Scientific Ethics, Aarhus approved the study, and the procedures were followed in accordance with the Helsinki Declaration.

### Cell lines

We analysed two bladder cancer cell lines RT4 (derived from grade I tumour) and T24 (derived from grade III tumour), obtained from the American Tissue Type Culture Collection (ATCC). Cells were cultured in McCoy's 5A modified medium, supplemented with 10% fetal calf serum, in T25 culture flasks (Nunc) in a humidified atmosphere containing 5% CO_2._ Cells were checked routinely for Mycoplasma infection (Mycoplasma PCR kit (Roche)). Cells were harvested at 90∼95% confluence, and washed twice with PBS (137 mM NaCl, 2.7 mM KCl, 10 mM Na_2_HPO_4_ and 2 mM KH_2_PO_4_, pH 7.4) and placed in a denaturing solution as described below.

### Preparation of total RNA

Tumour samples or cultured cells used for mRNA analysis were immediately placed in a denaturing solution (4 mol l^−1^ guanidine thiocyanate, 25 mmol l^−1^ sodium citrate (pH 7), 0.5% sarkosyl and 0.1 mmol l^−1^ 2-mercaptoethanol) and stored at −80°C. A frozen biopsy (<20 mg) was homogenised by a Heidolph Diax 600 mixer. Total RNA was extracted from tissues according to a slightly modified method of Chomczynski and Sacchi (1987). The RNA was resuspended in diethyl pyrocarbonate-treated double-distilled water and stored at −80°C. RNA was quantified using a UV spectrophotometer (*A*_260 nm_=1 corresponds to 40 *μ*g ml^−1^ RNA).

### Real-time RT–PCR quantification of mRNA

Quantification of mRNA was performed by real-time RT–PCR on the Lightcycler instrument (Roche). Primer sequences, annealing temperature and PCR products size for the components of the EGF family are presented in Table 2

**Table 2:** Primer sequences and primer conditions

**mRNA species (amplicon size)**	**Gene-specific primers (sense/antisense) (primer conc./annealing temperature)**	**External calibrator**
HER3 365 bp	5′-GGT GCT GGG CTT GCT TTT-3′ 5′-CGT GGC TGG AGT TGG TGT TA-3′ (5 pmol/65°C)	HEC
HER4 265 bp	5′-TGT GAG AAG ATG GAA GAT GGC-3′ 5′-GTT GTG GTA AAG TGG AAT GGC-3′ (5 pmol/65°C)	KLE
HRG1*α* 179 bp	5′-ATC CAC CAC TGG GAC A-3′ 5′-TTT GGA TCA TGG GCA-3′ (5 pmol/60°C)	KLE
HRG1*β* 321 bp	5′-TAG GAA ATG ACA GTG CCT C-3′ 5′-CGT AGT TTT GGC AGC GA-3′ (5 pmol/65°C)	KLE
HRG2*α* 308 bp	5′-AAA TAT GGC AAC GGC AG-3′ 5′-CGC AAA GGC AGT TTC T-3′ (5 pmol/60°C)	KLE
HRG2*β* 236 bp	5′-GCT TTA CGT CAA CAG CG-3′ 5′-CCG GTG TAT CCC ACA G-3′ (5 pmol/63°C)	HCV
HRG3 256 bp	5′-ACA GTG CAA GCG AAA AC-3′ 5′-CAC TAT GAT ATG AGG GCG-3′ (5 pmol/61°C)	KLE
HRG4 107 bp	5′-CTGTTGTCTGCGGTATTC-3′ 5′-TCATTCTTGGTCAAGAGAGT-3′ (5 pmol/61°C)	RT4
Beta-actin 313	5′-GGCGGCACCACCATGTACCCT-3′ 5′-AGGGGCCGGACTCGTCATACT-3′ (15 pmol/68°C)	KLE

. We conducted BLASTN searches to confirm the specificity of the nucleotide sequences chosen for the primers. Specificity was further verified by checking the size of the product by agarose gel electrophoresis and nucleotide sequencing using a 310 genetic analyser (Applied Biosystems) and BigDye Terminator Technology.

cDNA was generated in a reverse transcription reaction where 1 *μ*g RNA was mixed with 2.5 U AMW reverse transcriptase (Applied Biosytems) in a reaction mixture containing 10 mM Tris-HCL (pH 8.3), 1 U *μ*l^−1^ RNase inhibitor, 1 mmol l^−1^ deoxyribonucleoside triphosphate (dATP, dTTP, dGTP, dCTP), 2.5 *μ*mol l^−1^ 16-mer d(T)_16_ primer, 50 mmol l^−1^ KCl and 6.25 mmol l^−1^ MgCl_2_ in a total volume of 20 *μ*l (all reagents from Applied Biosystem). The reactions were incubated in a Perkin Elmer 9700 thermocycler for 90 s at 94°C followed by 30 min at 42°C and finally at 94°C for 1 min. This cDNA preparation was used for real-time PCR of all RNA species examined. Reverse transcription of all samples to cDNA was performed on the same day. Real-time PCR was performed with the Lightcycler Syber Green I quantification kit (Roche) in a total volume of 10 *μ*l in LC glass capillaries (Roche). Real-time PCR was conducted with the following profile: initial heating to 94°C for 90 s followed by 40–50 PCR cycles of heating to 94°C, incubation for 5 s at the annealing temperature specific for each mRNA species (Table 2) and incubation for 10 s at 72°C. The fluorescence data were collected and the mRNA quantified with lightcycler software version 3.3 by using the second derivative maximum method of quantification. A standard melting curve was used to check the quality of amplification. A calibration curve and positive and negative controls were included in each run. The calibration curve was composed of serial dilutions of a pool of culture cell lines RNA for each mRNA species (Table 2). To generate the calibration curve, calibrators with the following concentration of RNA were used: 1, 0.5, 0.25, 0.1, 0.05, 0.025, 0.01, 0.005 and 0.0025 *μ*g *μ*l^−1^. Positive control (in duplicate) was included in each run together with negative control, which was a sample without RNA added. As different calibrator are used for each gene and the expression is presented as relative expression to a specific calibrator, the levels of expressions are not comparable across different genes. Variation could exist in the amount of total RNA added to each reaction mix and in its quality. We therefore quantified transcripts of beta-actin as an endogenous RNA control, and each sample was normalised on the basis of its beta-actin mRNA contents. Beta-actin has been used as control gene in various studies on bladder cancer (Vageli *et al*, 1996; Chiu *et al*, 2002) as well as in breast cancer (Agudo *et al*, 2004). All the quantifications in this study are presented as the ratio between the target gene and beta-actin.

### Statistical analysis

Nonparametric tests were used throughout this study. Two-sided *P*-values less than 0.05 were considered to be significant. The Mann–Whitney *U*-test and Kruskal–Wallis test were used to compare the expression of the EGF family members with clinical stage, grade, tumour type and size of the tumour. Correlations were analysed using Spearman's rank correlation test. Life table calculations were performed using the Kaplan–Meier method. Comparison between the curves was carried out using log-rank test. (The software Graph Pad Prism (version 4) was used for statistical analysis.)

## RESULTS

### mRNA expression of HER3, HER4 and the HRGs

**Figure 1:**
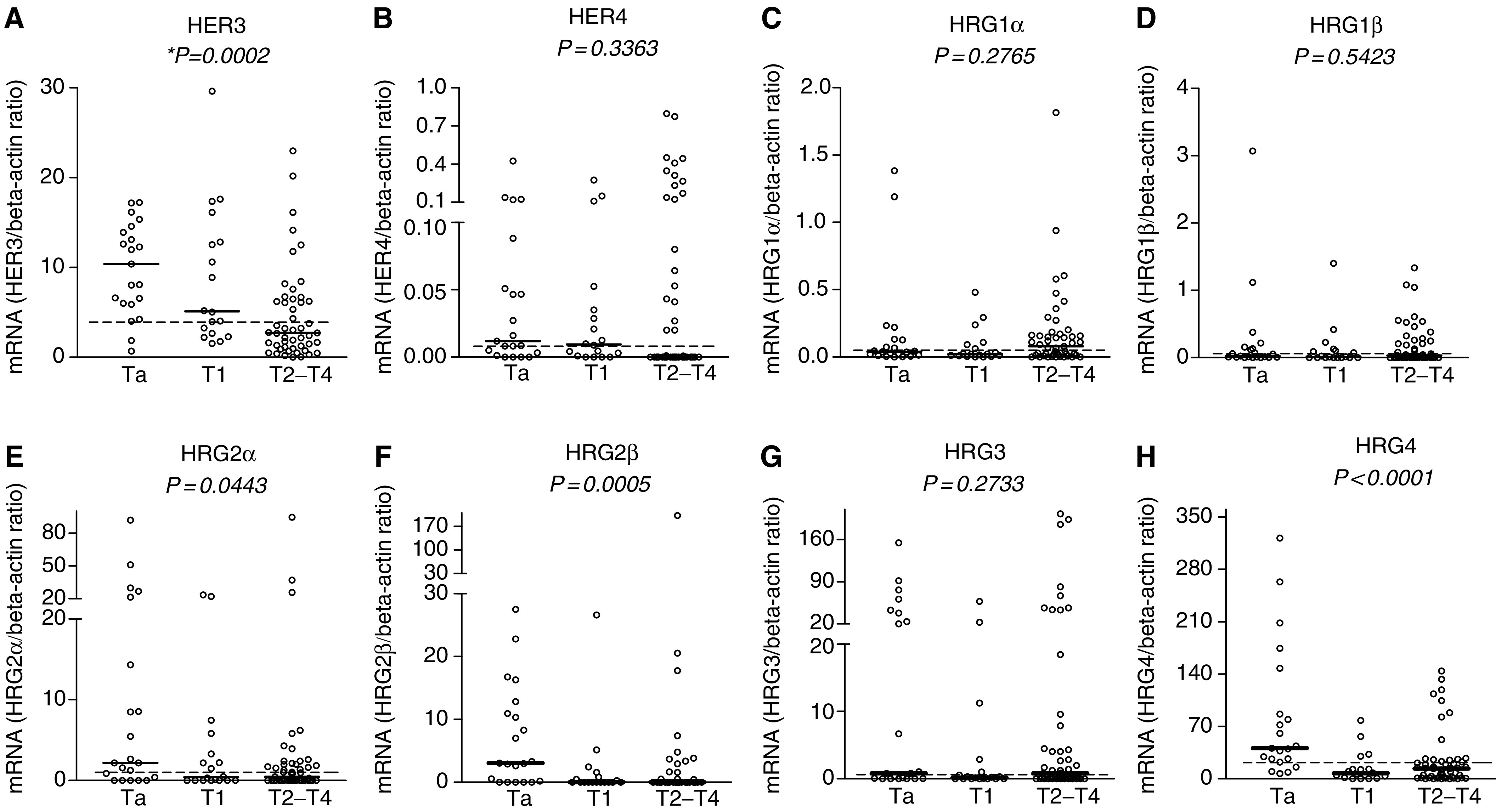
mRNA expression of HER3 (**A**), HER4 (**B**) and their ligands HRG1*α* (**C**), HRG1*β* (**D**), HRG2*α* (**E**), HRG2*β* (**F**), HRG3 (**G**) and HRG4 (**H**) in 88 human biopsy samples. Horizontal dashed lines show the median cutoff (median of all samples) used for survival analysis. Median cutoff for HRG2*β* was zero and samples were categorised as no HRG2*β* expression (low) or HRG2*β*-positive expression (high). Solid horizontal lines demonstrate the median of each individual group. All data are shown as the ratio between the target gene and beta-actin. ^*^P-value, nonparametric Kruskal–Wallis test.

The mRNA expression of two receptors, HER3 and HER4, and four ligands (HRG1–4), including the *α* and *β* isoforms of HRG1 and HRG2, was quantified in biopsies from 88 bladder cancer patients (Table 1) and two bladder cancer cell lines (RT4, derived from grade I; and T24, derived from grade III). Expression of the receptors HER3 and HER4, and ligands HRG1*α*, HRG1*β*, HRG2*α*, HRG2*β*, HRG3 and HRG4 was detected in 99, 63, 90, 61, 63, 43, 75 and 91% of the bladder cancer cases, respectively. The median concentrations of receptors were as follows: HER3 (median Ta (10.38), T1 (5.10), T2–T4 (2.72)) and HER4 (median Ta (0.012), T1 (0.009), T2–T4 (0.0)). The median concentrations of ligands were as follows: HRG1*α* (median Ta (0.04), T1 (0.022), T2–T4 (0.08)), HRG1*β* (median Ta (0.024), T1 (0.013), T2–T4 (0.03)), HRG2*α* (median Ta (2.22), T1 (0.41), T2–T4 (0.45)), HRG2*β* (median Ta (3.07), T1 (0.0), T2–T4 (0.0)), HRG3 (Ta (0.82), T1 (0.20), T2–T4 (0.81)) and HRG4 (median Ta (40.91), T1 (7.20), T2–T4 (13.76)) (Figure 1

). The differences were highly significant for HER3 (*P*⩽0.0002), HRG2*β* (*P*⩽0.0005) and HRG4 (*P*⩽0.0001) (Figure 1A, F and H). The expression level of HRG2*β* (*P*⩽0.001) and HRG4 (*P*⩽0.001) was also altered in biopsies classified as T1 in comparison with Ta (Figure 1F and H), which shows an early loss of HRG2*β* and HRG4 during bladder cancer progression. The median concentration of HER4, HRG1*α*, HRG1*β* and HRG3 did not show any significant difference among three groups (Figure 1).

**Figure 2:**
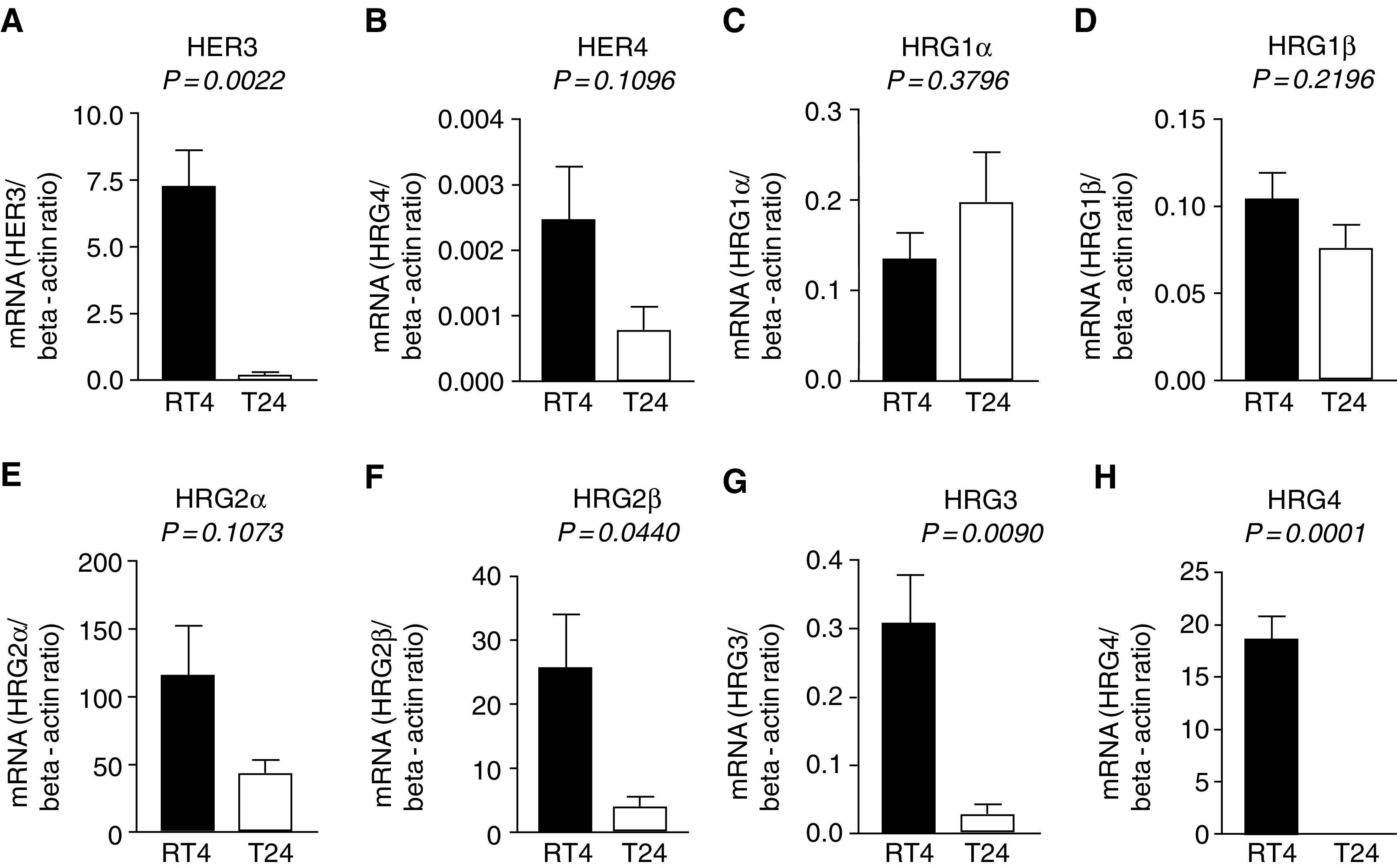
mRNA expression of HER3 (**A**), HER4 (**B**) and their ligands HRG1*α* (**C**), HRG1*β* (**D**), HRG2*α* (**E**), HRG2*β* (**F**), HRG3 (**G**) and HRG4 (**H**) in RT4 (derived from grade 1) and T24 (derived from grade 3) bladder cancer cell lines. Error bars represent standard deviation of at least four experiments. All data are shown as the ratio between the target gene and beta-actin.

Our results in two bladder cancer cell lines RT4 and T24, which have been used as models of superficial noninvasive and invasive tumours, respectively (Theodorescu *et al*, 1990; 1998; Redwood *et al*, 1992; Booth *et al*, 1997; Davies *et al*, 1999), show a similar pattern of mRNA expression as in bladder cancer biopsies, except for HRG3. The mRNA expression level of HER3 (*P*=0.0022), HRG2*β* (*P*=0.0440), HRG3 (*P*=0.0090) and HRG4 (0.0001) was significantly downregulated in T24 compared to RT4 (Figure 2A–H

). Only for HRG3, which shows no change in expression in biopsy samples, all other mRNA expression results in bladder cancer cell lines are in agreement with the results obtained from biopsies.

### Correlation with clinical and histopathological parameters

HER3 and HRG4 downregulation is strongly correlated with stage (T2–T4 compared to Ta), grade (III+IV compared to I) and type of the tumour (solid compared to papillary type) (Table 3

**Table 3:** Correlation between EGF family mRNA levels and clinicopathological parameters of bladder cancer

**Genes**	**Stage^a^**	**Grade^b^**	**Tumour type^c^**	**Size^d^**
HER3	0.0003^e^	0.0017	0.0002	NS
HER4	NS	NS	NS	NS
HRG1*α*	NS	NS	NS	NS
HRG1*β*	NS	NS	NS	NS
HRG2*α*	0.0159	NS	NS	NS
HRG2*β*	0.0007	0.0279	NS	NS
HRG3	NS	NS	NS	NS
HRG4	<0.0001	0.0016	0.0138	NS

a Ta *vs* T2–T4.

b I+II *vs* III+IV.

c Papillary *vs* solid type tumour.

d Tumour size <3 cm *vs* >3 cm.

e *P*-value (Mann–Whitney *U*-test); NS, not significant.

). Similarly, reduced expression of HRG2*β* is correlated with higher stage and grade of the tumour but not with tumour type. Finally, HRG2*α* mRNA level is only correlated with tumour stage. The mRNA expression of HER4, HRG1 (*α* and *β*) and HRG3 is not correlated with any of the clinical and pathological parameters of the tumours (Table 3).

### Inter-relationship between HER3, HER4 and their stimulating ligands

Using the Spearman's rank correlation test (Table 4

**Table 4:** Relationship between HER3, HER4 and their ligands, the heregulins

	**HER3**	**HER4**
HRG1*α*	−0.316^a^	−0.153
	0.0027^b^	NS (0.15)
HRG1*β*	−0.270	−0.145
	0.0109	NS (0.18)
HRG2*α*	+0.318	+0.237
	0.0026	0.0256
HRG2*β*	+0.290	+0.064
	0.0060	NS (0.54)
HRG3	−0.036	+0.082
	NS (0.73)	NS (0.44)
HRG4	+0.498	+0.473
	<0.0001	<0.0001

a Spearman's correlation coefficient.

b *P*-value, spearman's rank correlation test; NS, not significant.

), we found the strongest positive correlation between mRNA content of HER3 or HER4/HRG4 (*P*<0.0001) followed by HER3/HRG2*β* and HER3 or HER4/HRG2*α*. The strongest negative correlation was observed for HER3/HRG1*α* followed by HER3/HRG1*β*.

### Correlation with survival

We examined the correlation between survival and the expression of individual members of the EGF family. For this part of the study, the median concentrations of each mRNA examined from the bladder tumours were selected as the cutoff point dividing all the patients into two groups, one with high expression (above median) and another with low expression (below median). Finally, the prognostic significance of coexpression of receptors (HER3 and HER4) with their ligands (HRG1–4) was examined. Tumours were categorised into high and low groups using the same median concentration as cutoff (as described above). Only those tumours showing low (below median) or high (above median) mRNA level for both the receptor and ligand were included in this part of the analysis.

**Figure 3:**
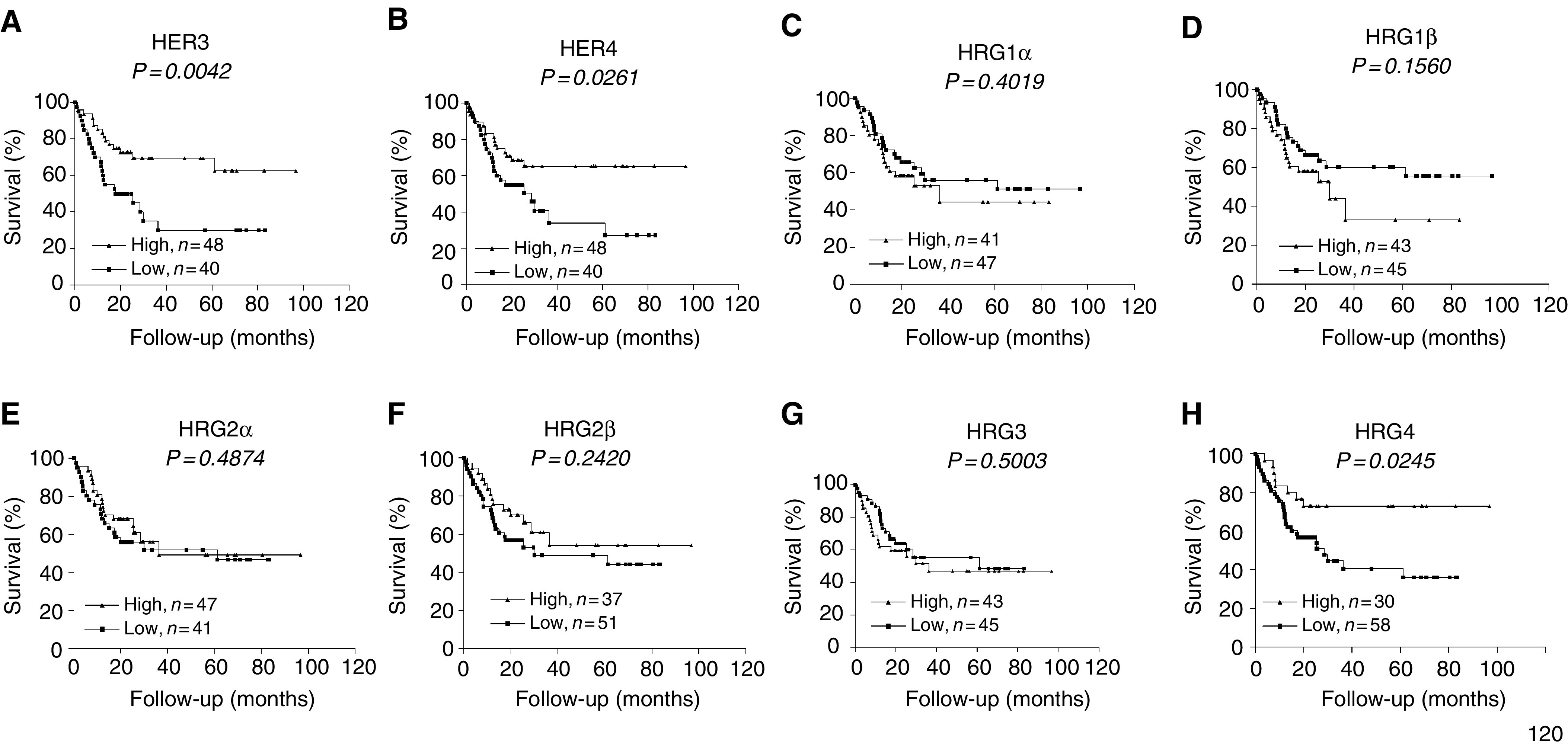
Kaplan–Meier survival curves for 88 bladder cancer patients plotted for HER3 (**A**), HER4 (**B**), HRG1*α* (**C**), HRG1*β* (**D**), HRG2*α* (**E**), HRG2*β* (**F**), HRG3 (**G**) and HRG4 (**H**). In all of the groups, the patients were categorised into low (below median) and high (above median).

**Figure 4:**
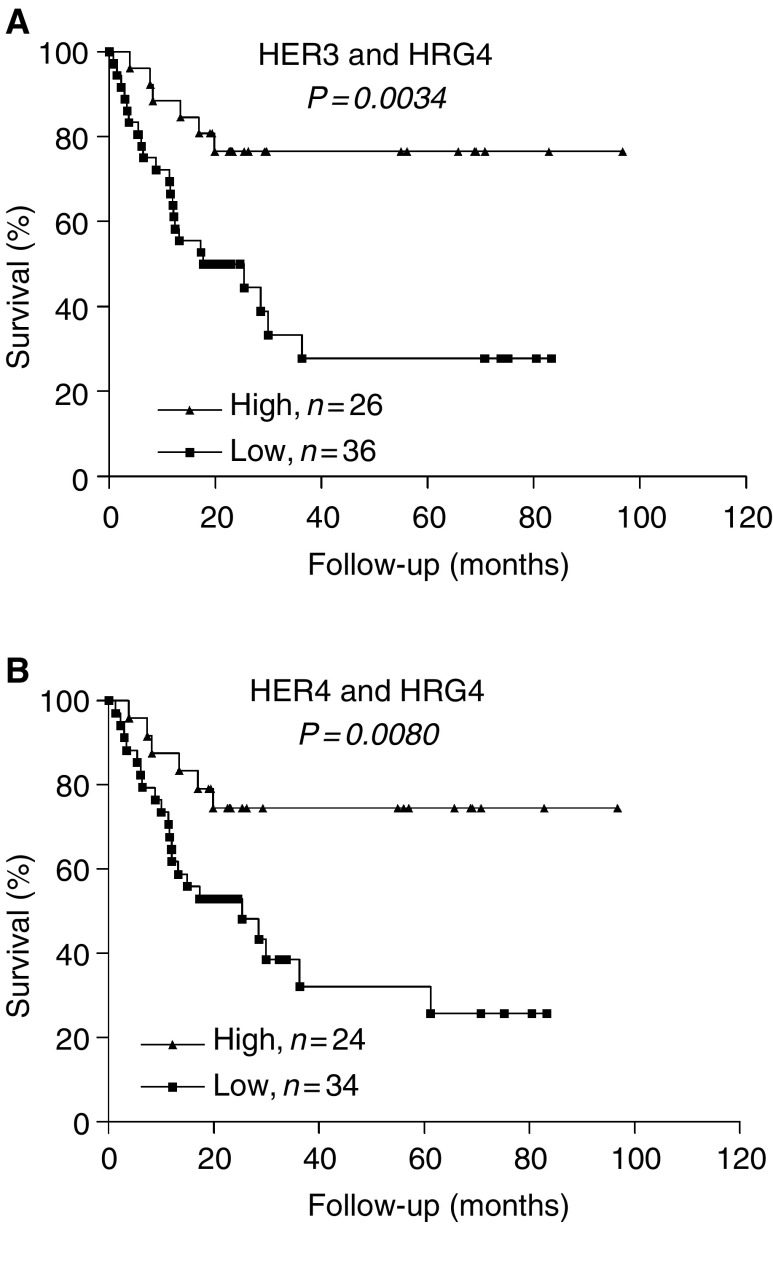
Kaplan–Meier survival curve for patient subpopulation coexpressing HER3–HRG4 (**A**) and HER4–HRG4 (**B**). Patients were categorised according to the low and high receptor/ligand mRNA coexpression.

Kaplan–Meier survival curves were made to evaluate the impact of ligand and receptor expression in context to survival of the patients (Figure 3A–H

). HER3 mRNA expression showed a highly significant correlation to survival (*P*=0.0042, *χ*^2^=8.18). Similarly, longer survival was observed in patients with elevated expression of HER4 (*P*=0.0261, *χ*^*2*^=4.948) and HRG4 (*P*=0.0245, *χ*^2^=5.062). Interestingly, coexpression of HER4 together with its stimulating ligand HRG4 (*P*=0.0080, *χ*^2^=7.04) showed even better correlation to survival than mRNA expression of HER4 alone (compare Figure 3B with Figure 4B

). In contrast to HER4, HER3 prognostic value was not greatly affected by HRG4 coexpression (compare Figure 3A with Figure 4A). No other receptor–ligand combinations correlated with the survival better than their expression alone. The HER4–HRG4 was found to be the best among all combinations analysed in this study.

## DISCUSSION

For the first time, data on the mRNA expression of all known HRGs and the HER3 and HER4 receptors, which they activate, are presented and related to the prognosis of bladder cancer patients. We have found an association between low HER3, HRG2*β* and HRG4 mRNA expressions and markers of tumour aggressiveness. More importantly, we have shown that HER3 and HER4 expression is a better prognostic indicator in bladder cancer patients than any other EGF family member analysed in this study, especially when coexpressed with HRG4.

The function of HER3 as a prognostic indicator is controversial based on the few studies performed on human tumours. Chow *et al* (2001) have suggested that the HER2 and HER3 expression profile is associated with favourable prognosis in bladder cancer patients, which is in agreement with the findings in the present study. In addition, HER3 expression is also associated with better prognosis in breast cancer (Pawlowski *et al*, 2000) and oral squamous cell carcinoma (Xia *et al*, 1999). In contrast, Witton *et al* (2003) have shown that high expression of HER3 is associated with poor prognosis in breast cancer patients. Discrepancies in the results of the few studies available so far justify further research into the role of HER3 in bladder and other cancers. However, our data support that high HER3 expression indicates better survival in patients with bladder cancer.

It is important to note that HER4 expression did not correlate with any of the clinical or pathological variables of the tumour. However, as mentioned before, its expression correlated with survival of bladder cancer patients, especially when coexpressed with HRG4. These results are consistent with the studies in bladder cancer (Chow *et al*, 2001) and most of the reports in breast cancer (Knowlden *et al*, 1998; Suo *et al*, 1998; 2001; Kew *et al*, 2000), showing HER4 expression to be a good prognostic indicator. In addition, studies of breast cancer cell lines have demonstrated that the antiproliferative and differentiation responses of HER4 are HRG dependent and correlate with HER4 activation (Sartor *et al*, 2001). HRG4 is a ligand for HER4 but not for the other three EGF receptors (Hobbs *et al*, 2002). Therefore, it is not surprising that HER4 when coexpressed with HRG4 shows significantly better prognostic value than its expression alone (Figures 3B and 4B). Therefore, it is important to consider expression of receptor as well as expression of the ligand when evaluating the final outcome of the disease and survival of patients. Our results suggest that combination of HER4–HRG4 can serve as a better indicator of survival than their individual expression and can identify a subgroup of patients who possibly may benefit from treatment directed towards the interaction between HER4 and HRG4.

Prognostic value of HER3 and HER4 was further analysed in samples from patients with tumours classified as T1–T4. We demonstrate that a high expression of HER3 correlates to survival and almost reaches significance (*P*=0.0887). Interestingly, a significant correlation with increased survival is found in patients with high expression of both HER3 and HER4 (*P*=0.0399) (data not shown). However, HER4 expression alone did not correlate significantly to survival in this group of patients (*P*=0.3125).

Recently, Junttila *et al* (2003) have analysed the expression of all four EGF receptors in bladder cancer cell lines and in bladder cancer biopsies. They have shown that the level of HER3 and HER4 was decreased in grade II (5637) and grade III (T24) bladder cancer cell lines, which is in keeping with our results on cell lines. In addition, they show that, compared to nonmalignant bladder samples, 50% of their 18 tumour samples express higher amount of HER3 and HER4. In addition, the study demonstrates that HER3 and HER4 mRNAs can reliably be quantified with real-time PCR. In the present study, we extend these results, and demonstrate a prognostic significance of the HER3 and HER4 receptors and their ligands, the HRGs.

HRG4 is relatively a new member of the EGF family and to our knowledge there is no report available showing any relationship between malignancies and HRG4. The HRG4 gene is distinct from the other three HRGs, suggesting a unique physiological role for HRG4 (Harari *et al*, 1999). Here, we present HRG4 as a novel prognostic marker for bladder cancer patients, especially when coexpressed with HER4. Additional investigations are required to elucidate the biological role of HRG4 and its relationship with other EGF family receptors and ligands.

We have noticed a significant loss of HRG2*β* mRNA expression in biopsies from invasive tumours (T1 and T2–T4) compared to superficial noninvasive tumours (Ta). However, no correlation was found between HRG2*β* expression and patient survival. HRG2*β* is a potent agonist for HER3 and HER4 (Hobbs *et al*, 2002). The impact of HRG2*β* in bladder cancer development and its prognostic significance is not known. However, by using MDA-MB-231 breast cancer cells, cocultured with endothelial cells, Nakano *et al* (2004) have shown that HRG2 is involved in an inhibitory effect on angiogenesis *in vitro* as well as *in vivo*. Therefore, it is possible that early loss of HRG2*β* during tumour development attenuates signalling through HER3 or HER4 and escalates cellular growth and angiogenesis, which ultimately leads to tumour progression and metastasis.

Our results indicate strong positive correlation between the mRNA expression levels of two receptors (HER3 and HER4) and ligands HRG2 and HRG4. There is also a negative correlation between HRG1 and HER3. These results concur with previous observations suggesting different roles for the HRGs. For example in MDA-MB-468 human mammary tumour cells, HRG2*β* inhibits cell growth, whereas HRG1*β* does not (Crovello *et al*, 1998). HRG1 also plays an important modulatory role in glioma cell invasion (Ritch *et al*, 2003). In addition, although not statistically significant, we have shown that high HRG1*β* expression correlates to poor survival in bladder cancer patients (Figure 3D), which is in accordance with recent findings in breast cancer showing that HRG1 is a key promoter of tumorigenicity and metastasis (Tsai *et al*, 2003). Together, these data suggest that HRG1 is functionally distinct from HRG2 and HRG4 and that the cell-type-specific effects of HER3 and HER4 resulting in proliferation or differentiation may relate to their stimulating ligands.

In order to explore whether the changes observed in bladder cancer biopsies were also reflected in cancer cell lines, we studied two bladder cancer cell lines representing grade 1 (RT4) and grade III (T24) tumours. Our results indicate that these cell lines have the same expression pattern as in biopsy samples except for one of the six HRGs studied, HRG3. These findings suggest that RT4 and T24 cells could be a useful model system for superficial and invasive tumours from bladder, respectively.

In conclusion, we report that HRGs are expressed in variable levels in bladder cancer and mRNA expressions of HER3, HRG2*β* and HRG4 are decreased in muscle-invasive tumours as compared to superficial tumours. Moreover, we report that increased expression of HER3, HER4 and HRG4 correlates to a better survival of patients with bladder cancer. Our results suggest that coexpression of HER3 and HER4 together with HRG4 shows better prognostic value than any of the other studied EGF family member alone.
